# Epidemiology of Pulmonary Nontuberculous Mycobacterial Disease, Japan^1^

**DOI:** 10.3201/eid2206.151086

**Published:** 2016-06

**Authors:** Ho Namkoong, Atsuyuki Kurashima, Kozo Morimoto, Yoshihiko Hoshino, Naoki Hasegawa, Manabu Ato, Satoshi Mitarai

**Affiliations:** Keio University School of Medicine, Tokyo, Japan (H. Namkoong, N. Hasegawa);; Japan Anti-Tuberculosis Association, Tokyo (A. Kurashima, K. Morimoto, S. Mitarai);; National Institute of Infectious Diseases, Tokyo (Y. Hoshino, M. Ato);; Nagasaki University Graduate School of Biomedical Sciences, Nagasaki, Japan (S. Mitarai)

**Keywords:** nontuberculous mycobacteria, NTM, epidemiology, incidence rate, nationwide survey, tuberculosis and other mycobacteria, Japan, bacteria, respiratory infections

**To the Editor:** Incidence of pulmonary nontuberculous mycobacterial disease (PNTMD) is reportedly increasing globally (*1*,*2*). Although such an increase is expected in Japan (*3*,*4*), the epidemiologic situation is unclear. The most recent survey, which used the 1997 American Thoracic Society diagnostic criteria, reported that the incidence rate for PNTMD in 2007 was 5.7 cases per 100,000 person-years (*5*). To update the data, we performed a nationwide hospital-based survey in Japan.

After a preliminary survey of 20 hospitals, we developed and disseminated questionnaires to all 884 hospitals in Japan that were certified by the Japanese Respiratory Society. The surveys asked about the number of newly diagnosed cases, from January through March 2014, of PNTMD, pulmonary *Mycobacterium avium* disease, *M. intracellulare* disease, or *M. avium* complex (MAC; the combination of the first 2 species listed); pulmonary *M. kansasii* disease; pulmonary *M. abscessus* disease; and tuberculosis (TB) for inpatients and outpatients. Hospital respondents returned the completed questionnaires by mail, fax, or Internet. To avoid potential reporting bias and misclassification, we counted only cases that met the 2007 American Thoracic Society/Infectious Diseases Society of America statements (*6*) and excluded cases diagnosed at other hospitals. Because the source population can be ascertained by using the epidemiologic data for TB as a reportable disease, to estimate the incidence rate of PNTMD, we used the ratio of TB to PNTMD cases. The PNTMD incidence rate was calculated as the national incidence rate of TB multiplied by the ratio of new PNTMD to TB cases reported by the responding hospitals (online Technical Appendix Figure 1, http://wwwnc.cdc.gov/EID/article/22/6/15-1086-Techapp1.pdf).

 To clarify the chronologic changes in incidence, we followed the same method for comparing TB and PNTMD used in a prior epidemiologic study in Japan (*5*). We established methods for maximizing survey response rates and facilitating ease of completion by offering extensive support to survey recipients (Technical Appendix Table 1).

We achieved a high response rate of 62.3% (551 hospitals), and in all regions the response rate exceeded 50% (Technical Appendix Table 2). The numbers of newly diagnosed cases were 2,327 for TB and 2,652 for PNTMD. Because the incidence rate for TB was reported to be 12.9 cases per 100,000 person-years, that of PNTMD was estimated to be 14.7 cases per 100,000 person-years, which is ≈2.6 times the incidence rate reported in 2007 (Figure). By using the same method, we found the incidence of pulmonary MAC, *M. kansasii,* and *M. abscessus* disease to be 13.1, 0.6, and 0.5 cases per 100,000 person-years, respectively (Technical Appendix Table 2). The ratio of pulmonary *M. avium* disease to MAC was higher in the northern and eastern parts of Japan, whereas the ratio of pulmonary *M. intracellulare* disease to MAC was higher in the southern and western parts of Japan (Technical Appendix Figure 1).

**Figure:**
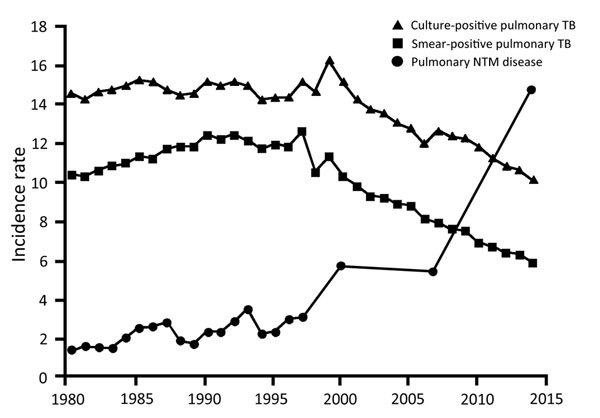
Incidence (no. cases/100,000 person-years) of pulmonary nontuberculous mycobacterial (NTM) disease, culture-positive tuberculosis (TB), and smear-positive TB in Japan during 1980–2014. The nationwide survey revealed that the incidence rate of pulmonary NTM disease exceeds that of TB. The epidemiologic survey before 1988 was conducted annually by the same research group; subsequently, another group performed the epidemiologic survey only in 2001 and 2007.

From this survey, we observed that the incidence rate of PNTMD may exceed that of TB and that incidence rates of PNTMD in Japan may be among the highest worldwide (Figure). This finding implies that the prevalence of PNTMD as a chronic infection is estimated to be much higher than that of TB.

We assume that the high rates of PNTMD in Japan are consistent with data suggesting that Asians are particularly susceptible to PNTMD (*1*,*7*,*8*). Other factors contributing to the increase might be the simplified diagnosis according to the 2007 American Thoracic Society/Infectious Diseases Society of America statements, increased awareness by medical staff, population aging, and increased frequency of medical checkups with computed tomography of the chest.

Another finding was the characteristic gradient clustering of the ratios of *M. avium* and *M. intracellulare* (Technical Appendix Figure 2). This finding supports the widely accepted belief that environmental factors strongly affect the epidemiology of PNTMD; therefore, the role of factors such as soil, humidity, temperature, and saturated vapor pressure should be seriously considered (*9*).

We also found dramatic increases in incidence of pulmonary *M. abscessus* disease and pulmonary MAC disease, whereas incidence of pulmonary *M. kansasii* disease was stable. Although we did not distinguish *M. massiliense* from *M. abscessus*, the incidence rate for pulmonary *M. abscessus* disease increased from 0.1 cases in 2001 to 0.5 cases per 100,000 person-years in 2014. This epidemiologic tendency should be monitored (*10*).

This study has several limitations. First, differing characteristics between the responding and nonresponding hospitals could cause bias. Second, we did not collect data outside of hospitals. Third, incomplete reporting could undermine the accuracy of our estimates (Technical Appendix Tables 3, 4). Therefore, the epidemiologic data should be verified by using other approaches (Technical Appendix Table 1). 

The dramatic increase in incidence rates for PNTMD warrants its recognition as a major public health concern. Because the prevalence rates of this currently incurable lifelong chronic disease are estimated to be high, the effect on the community could be enormous. Further investigations are needed.

Technical Appendix Incidence rates for mycobacterial infections in Japan during 1980–2014; response rate, results, characteristics, and limitations of survey of newly diagnosed pulmonary nontuberculous mycobacterial disease and mycobacterial disease, January–March 2014, Japan; and comparison of hospitals that did and did not respond to the survey.
