# Supplementary material for: Epidemiology of Pulmonary Nontuberculous Mycobacterial Disease, Japan
Source: Emerg Infect Dis. 2016 Jun;22(6):1116–7. doi: 10.3201/eid2206.151086 (PMC4880076; doi:10.3201/eid2206.151086)
Supplement: Technical Appendix — Incidence rates for mycobacterial infections in Japan during 1980–2014; response rate, results, characteristics, and limitations of survey of newly diagnosed pulmonary nontuberculous mycobacterial disease and mycobacterial disease, January–March 2014, Japan; and comparison of hospitals that did and did not respond to the survey. [file 15-1086-Techapp-s1.pdf]

Article DOI: http://dx.doi.org/10.3201/eid2206.151086

Epidemiology of Pulmonary Nontuberculous Mycobacterial Disease, Japan

Technical Appendix

Technical Appendix Table 1. Characteristics of this nationwide survey and other approaches for estimating the epidemiological data of PNTMD*

Multidisciplinary approaches for the accuracy of the epidemiological data in this nationwide survey*	
A	We conducted a preliminary survey of 20 hospitals before this survey. The aim of this preliminary survey was to identify points for improvement in the survey methods. We have discussed these points with the responders, and have modified the questionnaire contents to enhance understandability.
B	We strongly advised the responders to calculate the cases of newly diagnosed PNTMD by referring to an in-house bacteriological laboratory or external laboratory company because cases must be diagnosed in accordance with the 2007 ATS/IDSA statements. Specifically, the responders were advised to inquire about cases with respiratory specimens that are positive for NTM during the study period. Subsequently, they confirmed that these cases met the diagnostic criteria for the first time during the study period.
C	As another way to calculate the number of PNTMD, we recommended the use of the ICD-10 system. Under the Japanese health insurance system, an ICD-10 code should be assigned and the date of diagnosis should be documented in a patient's medical chart. We encouraged responders to use their own hospital's ICD-10 database to find newly diagnosed PNTMD during the study period.
D	To ensure adequate understanding of the responders, we disseminated this information through a Frequently Asked Questions document. We created a website for this survey to obtain precise information easily. We requested them to provide their e-mail addresses and phone numbers, and we directly contacted the responder if the data appeared doubtful.
E	The government (Ministry of Health, Labour and Welfare), the Japanese Respiratory Society, and the Japanese Society for Tuberculosis sent official documents requiring accurate Japanese epidemiological data to each hospital. These actions may have partially contributed to increasing the accuracy of our data.
Other approaches for estimating the epidemiological data of PNTMD	
A	Surveillance from laboratories that performed mycobacterial cultures.
B	Use of electrical medical records on PNTMD.
C	Utilization of postmarketing surveillance of antimycobacterial drugs.

*PNTMD: pulmonary nontuberculous mycobacterial disease; ATS/IDSA: American Thoracic Society/Infectious Diseases Society of America; ICD: International Classification of Diseases.

Technical Appendix Table 2. Response rate and results of survey of newly diagnosed pulmonary nontuberculous mycobacterial disease and mycobacterial disease, January–March 2014, Japan*

Region	Hospitals, no.	Response rate, %	NTM	*M. avium*	*M. intracellulare*	MAC	*M. kansasii*	*M. abscessus*	Others	TB
Hokkaido	34	58.8	74	48	6	12	3	1	4	45
Tohoku	61	55.7	147	101	33	5	2	2	4	58
Kanto	262	69.5	1,010	568	174	159	54	32	23	851
Chubu	145	58.6	435	257	115	25	5	13	20	400
Kinki	143	58.0	498	262	149	10	35	25	17	511
Chugoku	61	60.7	159	88	51	5	4	3	8	138
Shikoku	41	51.2	69	26	25	12	0	5	1	78
Kyushu	124	63.7	239	77	114	15	10	6	17	218
Okinawa	13	76.9	21	6	12	0	0	1	2	28
Nationwide	884	62.3	2,652	1,433	679	243	113	88	96	2,327

M., Mycobacterium, MAC: *Mycobacterium avium* complex. NTM, nontuberculous mycobacteria; TB, tuberculosis.

Technical Appendix Table 3. Limitations of this nationwide survey*

Study limitations	
A	Any differences of characteristics between the responding and nonresponding hospitals could cause a bias because the calculation method adopted in this study was based on the assumption that the ratio of PNTMD to tuberculosis is the same between these two groups. However, the apparent differences between the two groups were not admitted in terms of the number of beds, type of hospital (public vs. private), and whether the hospital is teaching or nonteaching (Appendix Table 3).
B	We did not collect data on tuberculosis and PNTMD outside hospitals. Regarding tuberculosis, primary care physicians tend to send suspected cases to hospitals with respiratory physicians in general, mainly due to the relatively high probability of tuberculosis in Japan. On the other hand, because of more complicated diagnostic criteria, we assume that a certain number of PNTM cases could be undiagnosed in the clinics since additional results of cultures may not be confirmed.
C	Incomplete reporting could undermine the accuracy of our estimate.

*PNTMD: pulmonary nontuberculous mycobacterial disease.

Technical Appendix Table 4. The comparison between responded hospitals and non-responded hospitals*

Variable	Responding hospitals (n=551)	Nonresponding hospitals (n=333)	P value
The number of beds mean ± SD (range)	409.5 ± 239.0	402.5 ± 226.3	0.178†
Type of hospital, no(%)	Public 364 (66.1%) / Private 187 (33.9%)	Public 224 (67.3%)/ Private 109 (32.7%)	0.713§
Teaching hospital, no(%)	404 (73.3%)	261 (78.4%)	0.092§

*SD: standard deviation

†: Student's t-test

§: χ2 test.

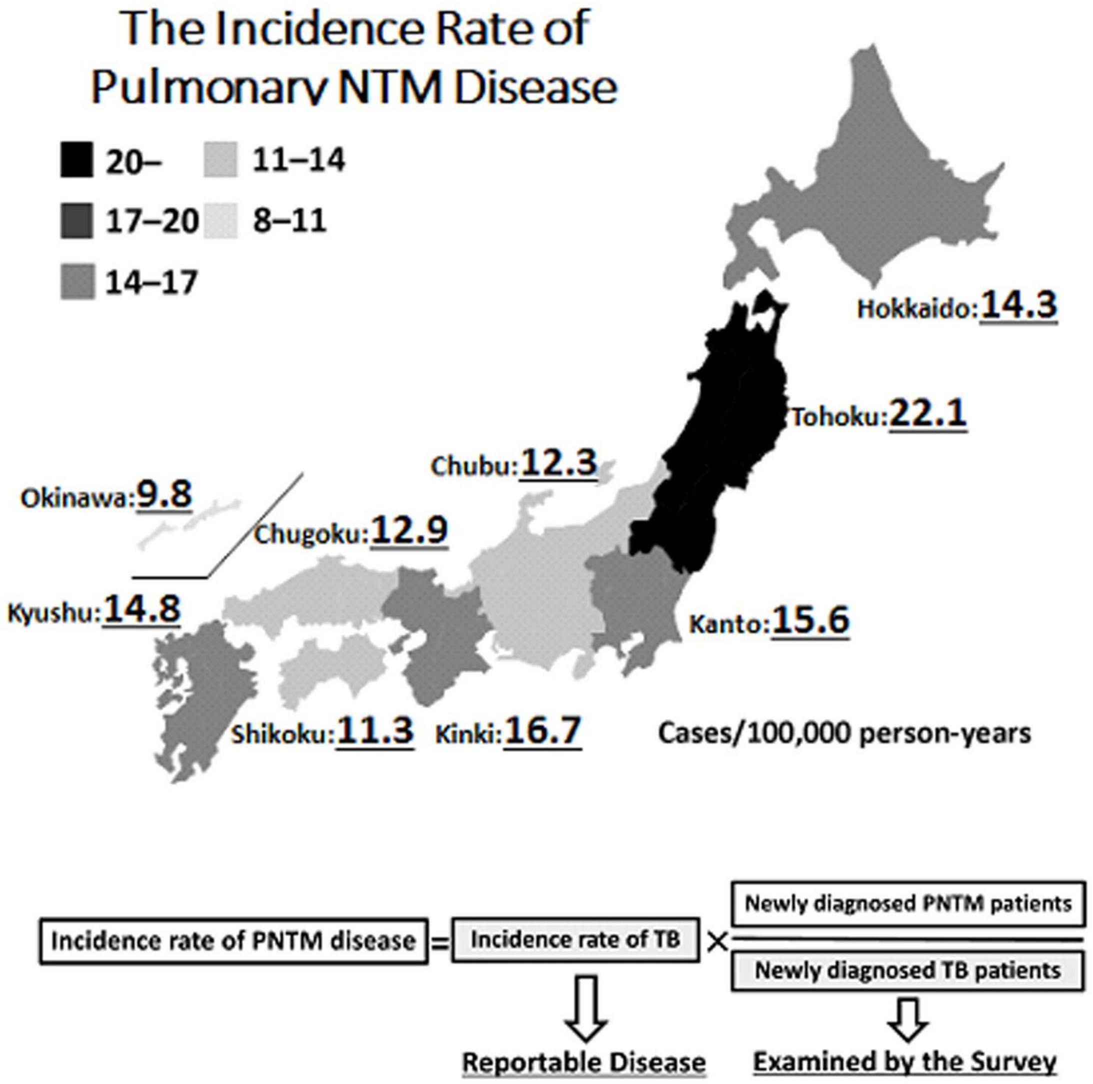

Technical Appendix Figure 1. The incidence rate of pulmonary NTM disease and pulmonary MAC disease are highest in Tohoku and lowest in Okinawa, the most southern part of Japan. The incidence rate of PNTMD was calculated as the national incidence rate of tuberculosis multiplied by the number of new PNTMD, divided by the number of new tuberculosis cases. NTM: Nontuberculous mycobacteria, PNTMD: Pulmonary nontuberculous mycobacterial disease.

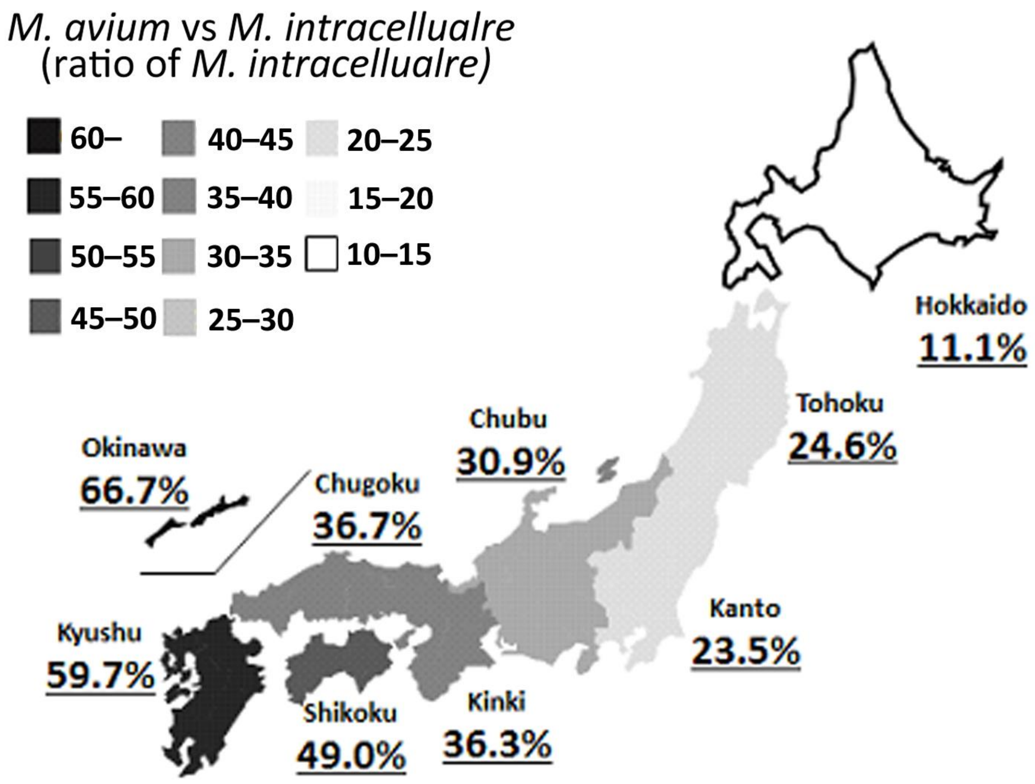

Technical Appendix Figure 2. Incidence of pulmonary *Mycobacterium avium* disease is higher in the northern and eastern parts of Japan, while that of pulmonary *Mycobacterium intracellulare* disease is higher in the southern and western parts of Japan. These findings are similar to those reported in the 2007 survey.
